# Supplementary material for: The interactome of intact mitochondria by cross-linking mass spectrometry provides evidence for coexisting respiratory supercomplexes
Source: Mol Cell Proteomics. 2017 Dec 8;17(2):216–32. doi: 10.1074/mcp.RA117.000470 (PMC5795388; doi:10.1074/mcp.RA117.000470)
Supplement: Supplemental Data [file supp_RA117.000470_133922_0_supp_23978_fzffwf.zip › spectra_annotation/mito_DR_spectra_annotation/359-1-1-1-4-1.pdf]

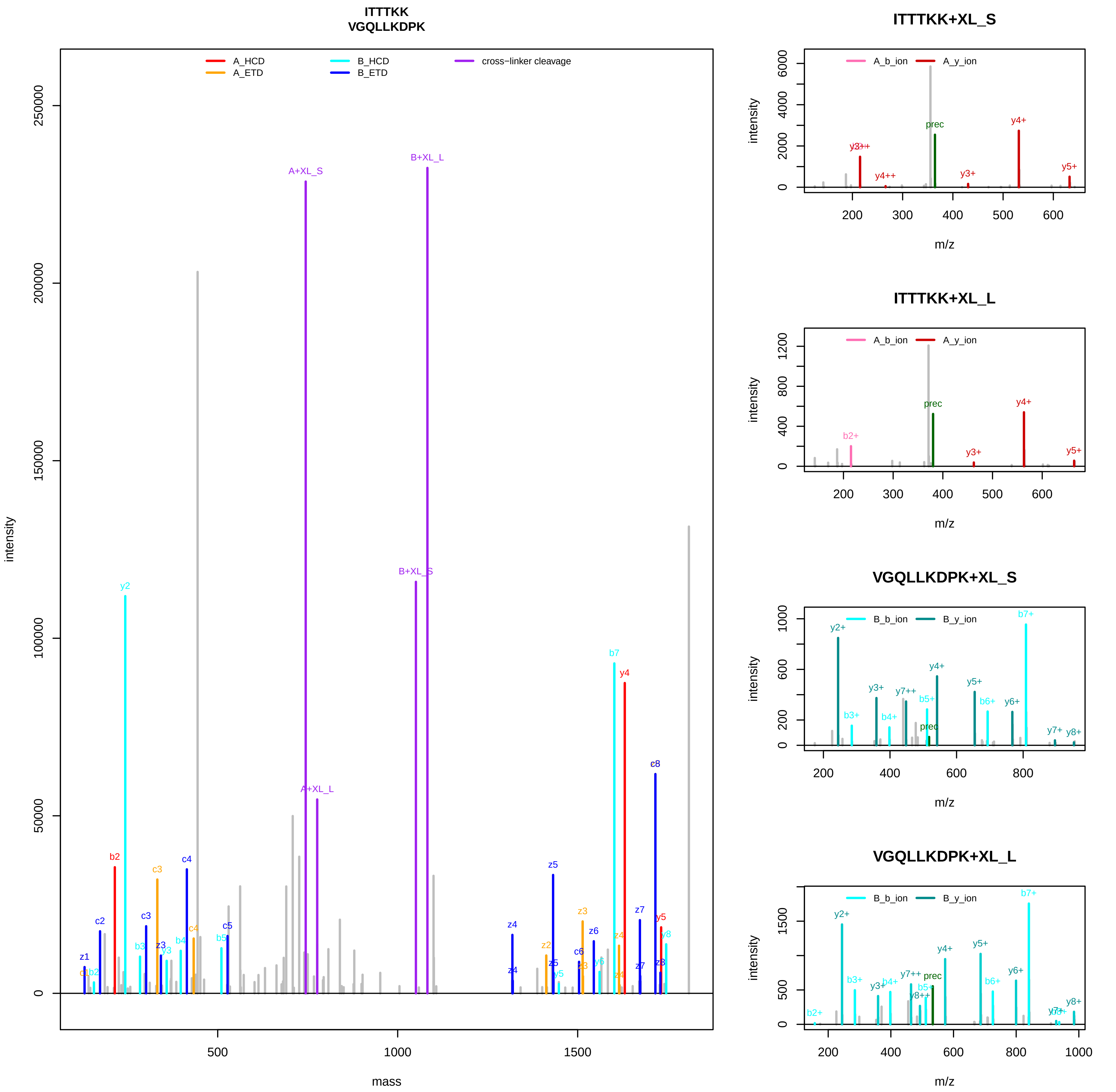

ITTTKK
VGQLLKDPK
A_HCD
A_ETD
B_HCD
B_ETD
cross−linker cleavage
intensity
mass
0
50000
100000
150000
200000
250000
500
1000
1500
A+XL_S
B+XL_L
B+XL_S
A+XL_L
y2
b2
c2
z1
c1
c3
b3
z3
y3
b4
c4
b5
c5
z4
z5
z2
z3
c6
z6
y5
y6
b7
y4
z7
c8
z8
y5
y8
ITTTKK+XL_S
A_b_ion
A_y_ion
y3++
y4++
prec
y3+
y4+
y5+
m/z
ITTTKK+XL_L
b2+
VGQLLKDPK+XL_S
B_b_ion
B_y_ion
y2+
b3+
y3+
b4+
y7++
b5+
y4+
y5+
b6+
y6+
b7+
y7+
y8+
VGQLLKDPK+XL_L
y8++
